# Microsoft Kinect-Based Artificial Perception System for Control of Functional Electrical Stimulation Assisted Grasping

**DOI:** 10.1155/2014/740469

**Published:** 2014-08-19

**Authors:** Matija Štrbac, Slobodan Kočović, Marko Marković, Dejan B. Popović

**Affiliations:** ^1^University of Belgrade-Faculty of Electrical Engineering, 11000 Belgrade, Serbia; ^2^Tecnalia Serbia Ltd., 11000 Belgrade, Serbia; ^3^Department of Translational Research and Knowledge Management, Otto Bock HealthCare GmbH, 37115 Duderstadt, Germany; ^4^Serbian Academy of Sciences and Arts (SASA), 11000 Belgrade, Serbia

## Abstract

We present a computer vision algorithm that incorporates a heuristic model which mimics a biological control system for the estimation of control signals used in functional electrical stimulation (FES) assisted grasping. The developed processing software acquires the data from Microsoft Kinect camera and implements real-time hand tracking and object analysis. This information can be used to identify temporal synchrony and spatial synergies modalities for FES control. Therefore, the algorithm acts as artificial perception which mimics human visual perception by identifying the position and shape of the object with respect to the position of the hand in real time during the planning phase of the grasp. This artificial perception used within the heuristically developed model allows selection of the appropriate grasp and prehension. The experiments demonstrate that correct grasp modality was selected in more than 90% of tested scenarios/objects. The system is portable, and the components are low in cost and robust; hence, it can be used for the FES in clinical or even home environment. The main application of the system is envisioned for functional electrical therapy, that is, intensive exercise assisted with FES.

## 1. Introduction

A functional electrical stimulation (FES) system comprises an electronic stimulator and surface electrodes as the interface for the delivery of bursts of electrical charge to motor systems in order to assist or generate function that is missing due to a neurological injury. Clinical studies suggested that intensive grasping exercise assisted with FES, termed functional electrical therapy (FET), in poststroke hemiplegic patients led to significant carryover effects [1, 2]. The FES system used in these studies assisted the opening and closing of the hand [3–5]. The control applied to FET is based on temporal synchrony and spatial synergies found to be appropriate for the generation of natural-like movement of a paralyzed arm/hand [6, 7]. Different types of grasping have characteristic temporal and spatial synergies that depend on the position of the object, its use once it is grasped, size, and shape. Natural-like movement resolves this by visual perception (vision and the experience in object manipulation developed through trial and error procedures while learning a skill) [8]. The object manipulation can be sequenced into the following phases: (1) move the hand to the object and orient/preshape it for the grasp (prehension), (2) grasp the object, (3) bring the object to the point of usage (e.g., mouth in order to drink), (4) use the object, (5) bring the hand to the point where the object will be released, and (6) release the object by opening the hand. Current state-of-the-art FES systems can mainly assist the distal movements of the paralyzed arm (fingers and thumb control, forearm and wrist rotations, and shoulder rotations to some degree). Ideally, electrical stimulation is applied over the motor system nerves which activate the desired forearm muscles, but in practical usage scenarios this is difficult to achieve. Multipad electrodes and asynchronous stimulation protocols [9, 10] are recent developments that improve the selectivity of the activated muscles and also contribute to the postponing of muscle fatigue [11, 12].

**Figure 1:**
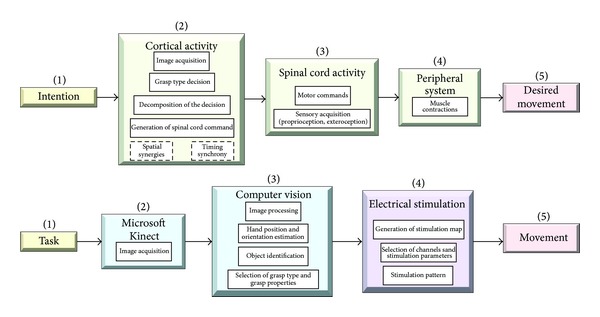
Models of the biological control (top) and artificial control for the functional electrical stimulation with the artificial perception system based on Microsoft Kinect (bottom). In a biological system based on intention (1) cortical activity (2) is transmitted over the spinal cord (3) to the peripheral system of interest (4) to produce the desired movement (5). In the envisioned artificial perception FES system, for a presented task (1) based on Microsoft Kinect data (2) computer vision algorithms (3) control the electrical stimulation parameters (4) in order to produce the movement (5).

The natural control of the prehension and grasping phases can be divided into three stages: decision, planning, and actuation. The hierarchical control model with the appropriate methods to represent the biological system is presented in Figure 1, top panel. The above-described FES does not include automatic decision based on perception and requires from the user to manually select the appropriate type of grasp. Consequently, the user has to concentrate on how to operate the device instead of paying more attention to the function that needs to be accomplished. This paper is suggesting a method that aims to decrease the cognitive burden by providing the following: automatic grasp type selection and automatic selection of the synergistic activation of various motor systems and stimulation parameters that are controlling contractions (Figure 1, bottom panel). The algorithm estimates hand orientation, aperture, trajectory, grasp type, and grasping timings used in the actuation phase. The automatic selection of muscles that generate the required prehension and stimulation parameters guaranties a stable grasp and safe use of the object for the desired function. Automatic control of this type requires an artificial perception system.

The selection of grasping modality has been approached in the history of FES by voice [13, 14], EMG control [15], brain-computer interface [16–18], and joystick [19, 20]. These systems can only accommodate for the automatic switch selection between two modalities of grasping [5] and proportional voluntary control of the intensity of stimulation.

Artificial perception was introduced for the control of grasping by a prosthetic hand in transradial amputees [21–23]. A similar system envisioned for use with a multipad electrode FES system and the related protocol are described in [24]. Tests on five healthy subjects showed that the software, which automatically decides the grasp type (lateral or palmar) and hand opening requirements in real time, demonstrated overall high recognition of the objects size and shape [25]. We have addressed the same problem by a custom stereovision system [26] that achieved reasonable performance in identification of objects and selection of appropriate grasp type. However, this system lacked information about the position and orientation of the hand with respect to the object of interest and could not achieve full functionality. 

We decided to use Microsoft Kinect [27] based on low-cost and computationally inexpensive gesture and voice recognition capabilities which proved to be robust enough to be utilized in many different engineering applications [28–30]. We demonstrated that Kinect can be used in an FES system as a sensory input for closed loop control of the forearm rotation in a tetraplegic patient during the reaching movement [31]. Kinect was also used as the component of the GO-SAIL electrical stimulation system [32, 33] where it captured and calculated shoulder and elbow joint angles based on dedicated Skeleton Tracking algorithm [34]. In GO-SAIL the estimation of the wrist and finger joint angles was based on electrogoniometers signals [32]. 

We present in the text that follows a method of processing data from Microsoft Kinect that outputs FES control signals. We also present how these control signals can be used within the heuristic model of grasping to create the decision of the grasping modality: (1) palmar, lateral, precision, or spherical grasp type, (2) hand aperture, (3) hand and forearm orientation, and (4) the timing of hand/forearm orientation and hand opening/closing. In our tests aforementioned data processing method resulted in more than 90% correctly selected movements (compared to healthy grasping) based on the generalized dataset of 25 objects in several different orientations.

## 2. Methods and Materials 

**Figure 2:**
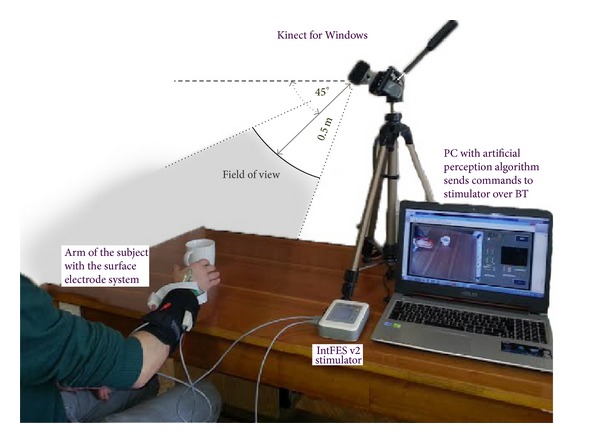
The envisioned artificial perception for control of FES assisted grasping system. PC uses image streams from the Kinect to generate a command concerning the type of grasping, size of the object, and relative position of the hand with respect to the selected object. The PC sends commands to the electronic stimulator (e.g., IntFES, Tecnalia, Spain) via Bluetooth. The stimulator interfaces motor systems of a patient by electrodes (e.g., multipad electrode array, Tecnalia, Spain) that are appropriate for the safe and stable operation of grasping, manipulation, and release of the object.

The Kinect sensor comprises (1) an RGB camera that captures 640 × 480 resolution images with 30 frames per second and (2) an infrared depth sensor. The output from Kinect provides two corresponding image streams: an RGB image with 32-bit resolution and a grayscale depth image with 11-bit resolution [35]. When Kinect is set to work in the “*near mode,*” the grayscale image represents depth data in the range of 500 mm to 3000 mm from the camera. To optimize the working area and the viewing angle of the camera, Kinect was mounted on a 0.6 m high camera stand. The camera stand was posted on the surface of the working table (lateral to the side of the user), and Kinect was tilted at 45° with respect to the surface of the table (transverse plane) as shown in Figure 2. This system setup at the same time ensures simple integration of the Kinect into the FET environment. 

Kinect was connected to a PC (via USB interface) and through dynamically linked Kinect SDK [36] subroutines. In order to achieve better performance and faster computation, most of the image processing was mapped onto the graphics processing unit (GPU) [37], while sequential algorithms were handled by the CPU. 

*Background Estimation. *The area above the table is defined as the working zone, described by the background image (BCG). BCG can be estimated during a short time-frame (*t *< 1 s) at the start of the program execution or loaded from a previously recorded session. Estimation of the real world coordinates in the depth image matrix is done by mapping the depth values of each pixel from the depth image with the corresponding 3D coordinates [38] with a calibrated Kinect sensor. Pixels that belong to the table can be extracted from these coordinates with a mathematical model based on table geometry, provided that this model is explicitly defined. Under the presumption that, in the presented system setup, the table will occupy more than 60% of the scene (from the camera viewpoint) we defined this mathematical model as the largest plane in the image.

Robust estimation of the model parameters based on defined geometry in computer vision applications is commonly solved with Random Sample Consensus (RANSAC) algorithm [39]. Basic RANSAC algorithm for plane detection would consist of randomly selecting* N* triplets of points from 3D image data, modeling* N* planes from these triplets, and counting inliers, that is, the pixels belonging to each plane. In order to achieve real-time performance, we have proposed an optimization step which consists of forming plane clusters, based on the angle between their perpendicular vectors, and counting the inliers only for the planes that belong to the largest cluster. Faster calculation with equivalent performance can be achieved through this intermediate clustering step [40].

**Figure 3:**
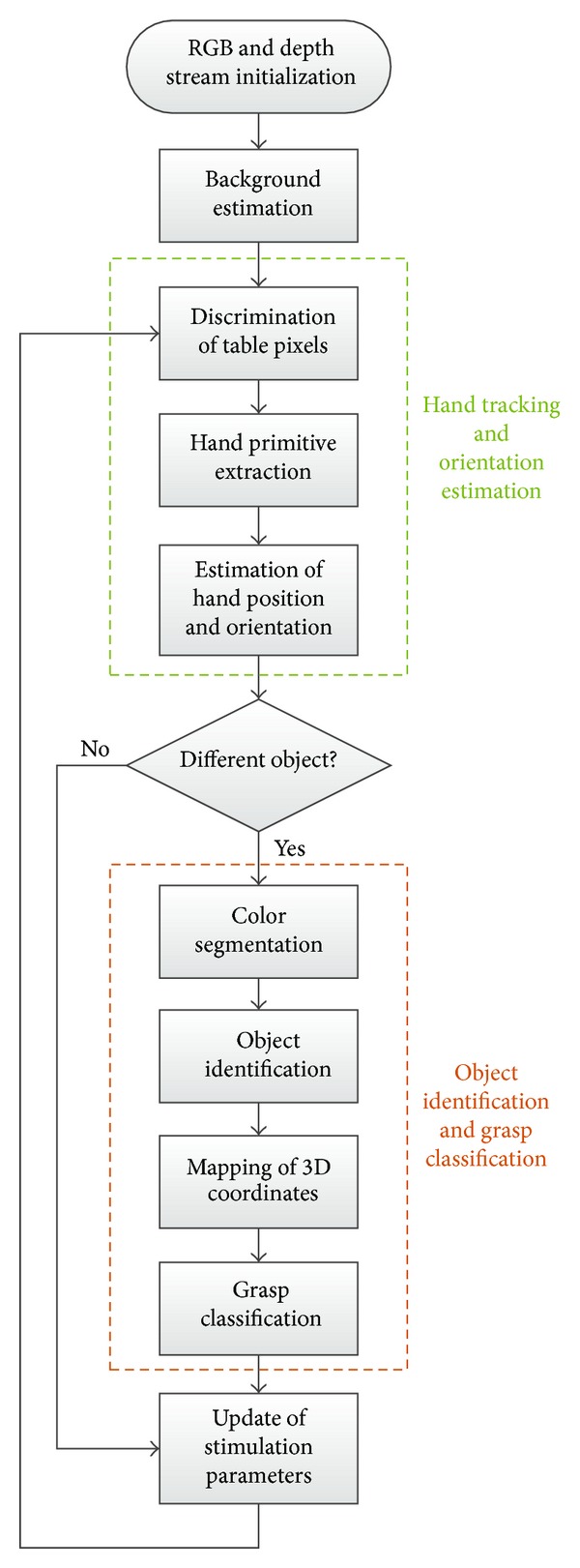
Computer vision algorithm for automatic control of FES based on Kinect data. Background is formed at the start of program execution; hand tracking and orientation estimation are performed in real time based on Kinect depth data, and object identification and grasp classification algorithms are executed when the object selection is changed based on RGB-D data.

The background image is formed by padding the closed contours (formed by objects that are on the table) and averaging the first* N* (where* N* is empirically set between 10 and 20) frames of the previously described RANSAC algorithm outputs. After BCG is estimated, the artificial perception algorithm starts to operate. This algorithm can be divided into two main parts: (1) hand tracking and orientation estimation and (2) object extraction and grasp type classification (Figure 3). Hand tracking with hand orientation estimation is done in real time based on the depth image stream. Object primitive extraction and grasp type classification use the RGB-D data and are executed at discrete time events (e.g., only when the object is removed and changed for another).

### 2.1. Hand Tracking

*Discrimination of Table Pixels*. The optical flow algorithm [41] was inadequate choice for hand tracking because our system should work continuously in both static and dynamic conditions, that is, when the hand is above the table but is not moving, and at the same time allow manipulation and change of objects and their position. In order to obtain the information about hand position in the depth image we used RANSAC discrimination of table pixels, this time while the subject had his/her hand above the table. 

*Hand Primitive Extraction. *We assumed that the arm of the subject is intersecting the table edge. This intersection was detected by filtering the BCG through the low-threshold Sobel edge filter and modeling the two longest edges in the filtered image with the corresponding line models based on their coordinates. Subsequently, the edges of the table are detected from the depth image for every frame in real time, and these edges are matched with the equations of the line models from the BCG. When the subject is preparing for grasp and their arm is over the table, the extracted table edges will feature a gap at the location of the arm. As a further verification whether the identified gap represents the human arm and is not a consequence of errors in morphological operations, the mean depth of the surrounding pixels on one side and the other side of the table edge is compared. If both arms are above the table, the one closer to the camera will be selected, implying that in FES applications the camera should be lateral to the paretic arm.

**Figure 4:**
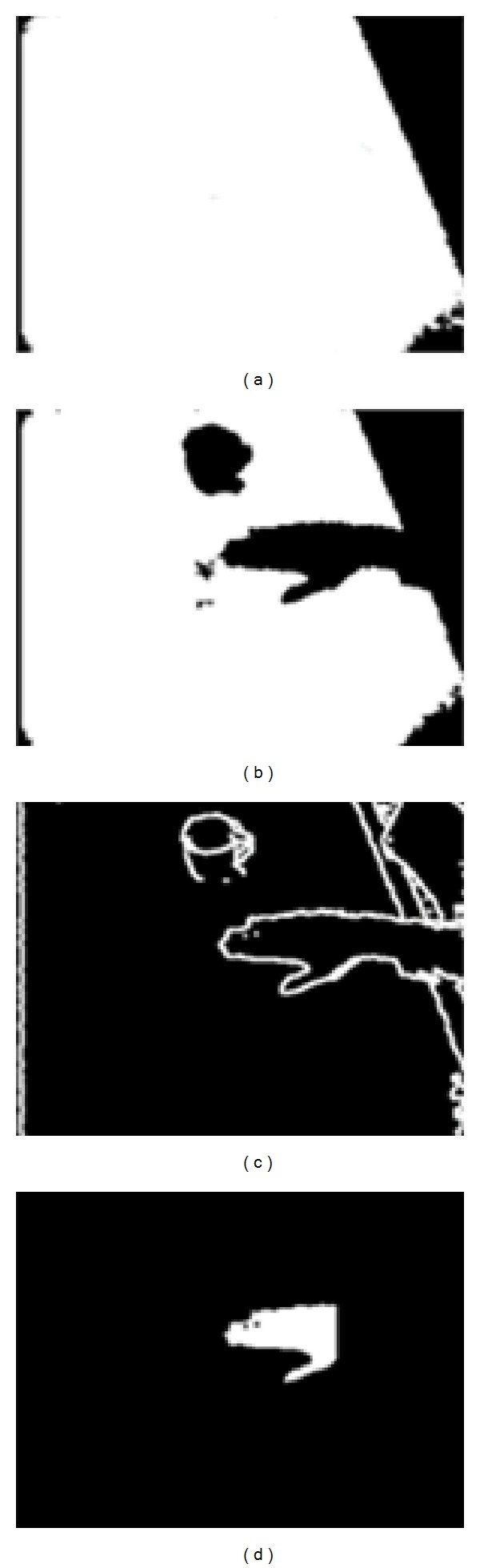
Several stages in the hand tracking algorithm: (a) background image; (b) table pixels during hand movement; (c) low-threshold SOBEL edge filtered depth image; (d) extracted hand primitive.

Image primitive of the arm is extracted by performing image fill operation on the Sobel filtered depth image starting from the central pixel of the identified gap. The starting point of the arm is defined as the point that is not above the table and is most distant from the table edge, based on the 3D coordinates of the extracted primitive. The pixel on the fingertip is identified by calculating the furthest point from the starting point of the arm. All pixels belonging to the hand are extracted by recruiting points in the heuristically defined area around the fingertip pixel of the arm primitive (Figure 4). 

*Estimation of Hand Position and Orientation. *The median pixel of the hand primitive is used as the hand reference point and the coordinates corresponding to this pixel are used for real world position estimation in the hand tracking algorithm. If the hand is opened, this reference point will be placed on the area belonging to metacarpals or proximal phalanges, corresponding to the center of the grasp. Orientation of the hand is estimated from the real world coordinates of hand pixels in the depth image. Based on these points, using a RANSAC algorithm, the hand is modeled with a matching plane equation. From the cross product between the perpendicular vector of the hand plane and the perpendicular vector of the table plane, the spatial angle of the hand is estimated. Information about hand pronation/supination and wrist flexion/extension is gathered by using the dot product of the selected vector projections on the plane of interest. The accuracy of these estimates is in the range of 15 degrees, which is more than sufficient to achieve a fully functional grasp, as patients can compensate for these small errors with body movements. It is important to notice that the plane model was chosen because, in the process of grasping, finger extension is always performed prior to hand closure, that is, before the appropriate finger flexion sequence is performed. During this period of stimulation, while the fingers are extended, information about the hand orientation can be used in computer vision controlled FES system for adjusting the stimulation parameters and timely triggering of the stimulation sequence.

### 2.2. Object Identification with Grasp Classification

*Color Segmentation*. The minimum size of objects that can be detected by padding closed contours in the table primitive is confined with the threshold of RANSAC algorithm for table detection. It is important to notice that, with this approach, small objects that are lying on the table (e.g., pen, toothbrush, and keys) cannot be identified. For this reason, we have implemented another approach that relies on the color segmentation of the RGB image and mapping of the real world coordinates. With a color segmentation algorithm [42], the pixels of the RGB image that correspond to the user defined range of values of the red, green, and blue color components can be identified. Whereas a table takes more than 50% of the image, table color template can be defined by calculating the median value of all the pixels in the RGB image. All objects that are lying on the table are identified by padding of the closed contours in the table template, calculated through color segmentation, and then subtracting the template before the padding. Hereby, we have formed an algorithm which is not sensitive to the size of the object, but its color, therefore requiring that the objects used in the exercise must be of a different color than the table. Changing the threshold of deviation from this median color in the segmentation algorithm defines the tradeoff between indifference to table color patterns and responsiveness to object color. 

*Object Identification. *From objects that are extracted with the table template segmentation, our algorithm will select the largest object for grasping. This cost function for object selection can be easily changed; that is, the object closest to the hand of the subject can be selected by the algorithm. After cropping the area with the selected object from the color image, a primitive that contains only the object of interest is forwarded to the 3D mapping algorithm. 

*Mapping of 3D Coordinates. *Information about the 3D coordinates is coded in the depth image. Due to a different position of the RGB camera and the IR sensor, resulting in a different perspective in the depth and RGB images, joint calibration of RGB and depth images is required to use the object 3D information for classification. The aforementioned depth camera calibration [38] results in optimal estimates of the real world coordinates from the measured disparity units. RGB and depth camera system calibration requires a more complex model to correct the depth data and enable mapping of depth information to the color image. This calibration process was performed based on the procedure described by Daniel Herrera et al. [43]. At the distances used in the described system setup, the calibration of the Kinect cameras resulted in twice the reconstruction accuracy compared to the manufacturer calibration. 

Since 3D coordinates of an object are calculated for the camera reference system with the origin point in the center of the depth image sensor, translation and rotation of the reference system are needed in order to determine the height, the width, and the length of the object. Any point belonging to the table can be selected as a new origin point. Rotation angle should be determined by the camera angle with respect to the horizontal plane, that is, 45° for the defined system setup. New coordinates of the object are estimated by translating the coordinates from the camera reference system to the new origin point and then rotating the coordinate system so that the* z*-axis represents the vertical component, that is, height of the object:

(1) newCoordinates=[oldCoordinates−newOriginPoint] ×[010sin⁡θ0−cos⁡⁡θ−cos⁡⁡θ0−sin⁡θ].

The extracted object primitive and the 3D coordinates of the extracted object in the new reference system are forwarded to the algorithm for grasp type classification. 

*Grasp Classification. *We wanted to classify the objects used in activities of daily living to one of the four grasp types that can be achieved with electrical stimulation. Based on Kinect images of a closed set of objects that included a bottle, a mug, a coffee cup, a pencil, a spoon, a cell phone, a CD, and a ball we have formed a training database (30 images of 8 objects) for adjusting the classification parameters. The correct grasp for each object in the database was identified using the experiment illustrated in [26] on 8 healthy volunteers. The results of this experiment are presented in Table 1. It should be noted that for a mug and a coffee cup, the correct grasp type is ambiguous because it depends on the object orientation. In the training dataset, this was represented by images of these objects in different orientations. This training database of images was used for optimization of several thresholds in the sequential classification algorithm.

At the beginning of the classification procedure, the objects are divided into two groups based on their height: small objects that are lying on the table and can be grasped with lateral or pinch grasp and large objects that can be grasped with lateral, palmar, or spherical grasp. In the optimization process based on the training dataset, the object height threshold was defined empirically (4 cm). For small objects the ratio between the minor axis and major axis length of the ellipse that has the same normalized second central moments of the extracted object primitive is used for classification. Based on the images in the training database the classification threshold was optimized. The optimization resulted in the first rule of classification.* Rule (1).* If the difference in length between the major axis and the minor axis of the ellipse for a small object is greater than 60%, lateral grasp is selected; otherwise, pinch grasp is selected.

**Figure 5:**
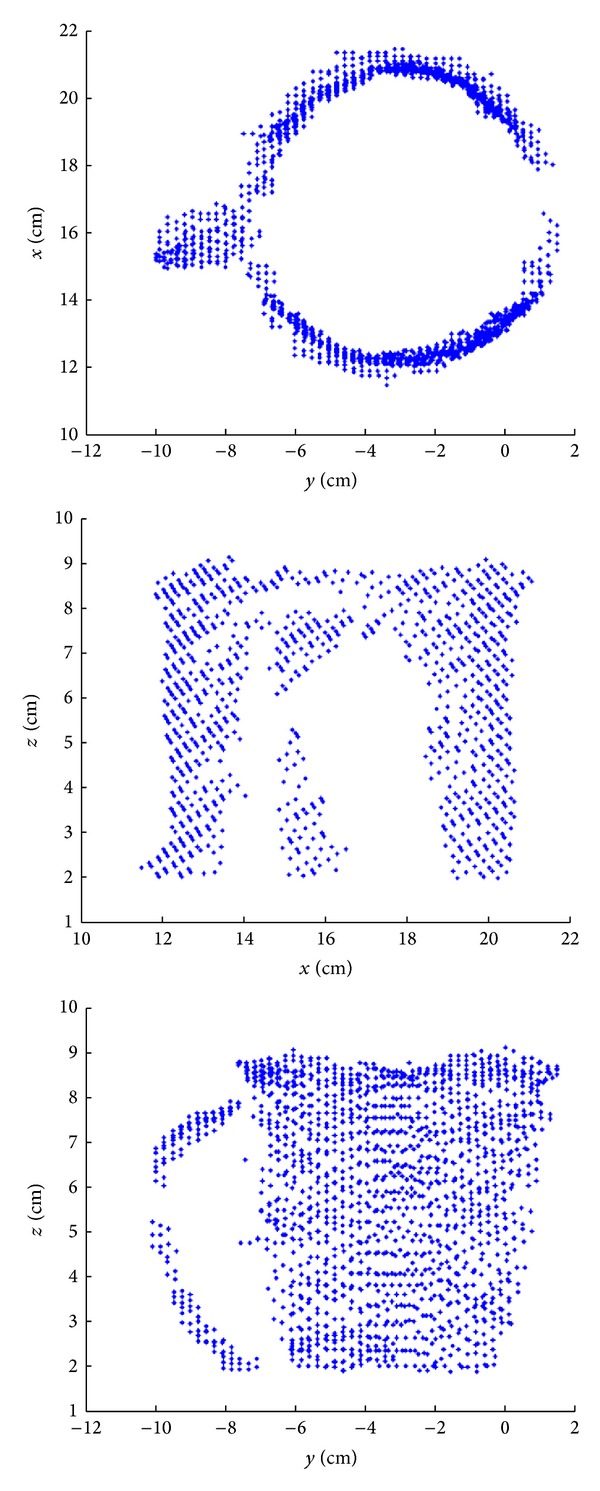
Projection of the 3D object coordinates on the transversal, frontal, and sagittal plane. The handle is clearly visible on the sagittal plane projection, and it can be easily detected with a few simple morphological operations. Handle orientation can be detected from the transversal projection. The existence of the handle and the handle orientation are used for classification of large objects.

For the classification of large objects in the first-phase, spherical grasp is identified as a grasp used only for round objects, which can be easily distinguished by forming the same ellipse model from the extracted primitive. Optimization of this classification threshold on the training dataset resulted in the second rule of the rule-based classifier.* Rule (2).* If length difference between the major axis and the minor axis of the ellipse for large object is less than 5%, spherical grasp is selected. For large objects information about the projection of the 3D coordinates (Figure 5) of the extracted object primitive onto the transversal, frontal, and sagittal plane is needed by the classifier to select palmar or lateral grasp.

In this stage, all points close to the table plane are excluded from the object model because large objects can cast a shadow that will sometimes be considered part of the object in the color segmentation extracted primitive. To obtain the projection of the object coordinates on the plane of interest we first calculate the perpendicular vector of the plane with the following equation:

(2) planeVector=([100]×[010sin⁡(az)0−cos⁡⁡(az)−cos⁡⁡(az)0−sin⁡(az)]) ×[cos⁡⁡(el)0sin⁡(el)010−sin⁡(el)0cos⁡⁡(el)],

where the angles “az” and “el” represent azimuth and elevation angle of the defined projection view. These angles change with the observation plane as follows:

- for the transverse plane: az = 0° and el = 90°,
- for the frontal plane: az = 90° and el = 0°,
- for the sagittal plane: az = 0° and el = 0°.

The set of object coordinates in the observation plane is calculated using the following formula:

(3) planeProjection=objectCoordinatesT ×projectionVector,

where projectionVector is the orthogonal basis of the planeVector matrix calculated through singular decomposition.

Projection used for classification is selected depending on the orientation of the observed object with respect to the camera and the hand of the subject. As the lateral grasp is used for large objects only when there is a handle on the object, from the projection of the object on the transverse plane, the algorithm determines whether the observed object has a handle. For objects with a handle, information about the handle orientation is acquired from the frontal and sagittal plane projections of the extracted object primitive.* Rule (3).* If the handle is detected on the projection and is from the same side as the hand of the subject, lateral grasp is selected; otherwise, palmar grasp is selected. For objects with no handle, palmar grasp is selected automatically, and orientation information is used only for aligning the hand with the longer axis of the object during prehension.

*Update of Stimulation Parameters. *IntFES stimulator (Tecnalia Research and Innovation, San Sebastian, Spain) can store custom, user-defined stimulation patterns that can be activated via Bluetooth protocol. After initial calibration of the multipad electrode [9], time sequence for changes of electrode configuration, that is, combination of active pads on the electrode, that will result in palmar, lateral, pinch, and spherical grasp can be defined. These stimulation patterns define activation timings of the electrode pads that will result in adequate muscle response and the grasping force. During functional electrical therapy, these predefined sequences can be activated in real time with the corresponding Bluetooth command as illustrated in the hybrid brain-computer interface system [18]. The computer vision system will, in addition to the adequate grasp type, be able to recognize the size and orientation of the object and position and orientation of the affected hand, thus enabling fine tuning of stimulation amplitude during hand opening and timely triggering of grasping stimulation pattern. After the object is grasped, the stimulation pattern for releasing the object will be automatically activated after a predefined amount of time (where the amount of time can be interactively defined for each object type). Upon the object release, the control loop restarts.

### 2.3. Experimental Setup

*Testing Image Dataset. *We have evaluated the developed artificial perception algorithm on a set of 25 different objects. These objects include two balls (different colors and sizes), a mobile phone, keys with and without a keychain, two mugs (different colors and shapes), a coffee cup, a coffee pot, two pencils, a spoon, a bottle, a flash drive with a USB connector, and a CD case. In order to evaluate the algorithm, we have formed a testing database with 86 images containing objects in various orientations, which resulted in the following:

- 42 images for lateral grasp (coffee cup, handle visible; mug, handle visible; coffee pot; pencil; and spoon),
- 17 images for palmar grasp (coffee cup, handle hidden; mug, handle hidden; bottle; and CD in a vertical orientation),
- 19 images for pinch grasp (mobile phone, keys, flash drive, and CD in a horizontal orientation),
- 6 images for spherical grasp (smaller and larger ball).

The system decisions, for this image set, were then compared against the results obtained from the recorded grasp patterns in eight able-bodied subjects (as described in [26]).

*Real-Time Evaluation. *We tested the system responsiveness in a real-time task on a single able-bodied subject in the position shown in Figure 2, but without the stimulation system. The outputs of the artificial perception algorithm used to update stimulation parameters were monitored, while the subject interacted with several different objects, and algorithm processing time was evaluated.

## 3. Results 

**Figure 6:**
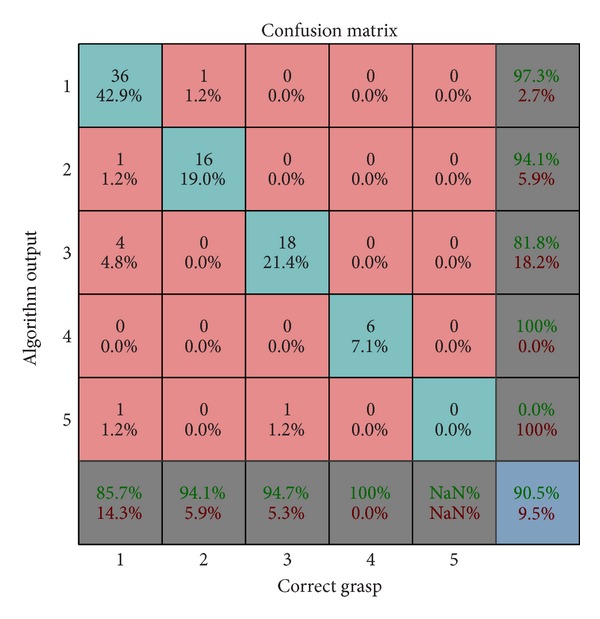
Confusion matrix for the grasp classification on the testing dataset: (1) lateral grasp, (2) palmar grasp, (3) pinch grasp, (4) spherical grasp, and (5) faulty object extraction. Overall results imply that the proposed classification algorithm will select the adequate grasp for more than 90% of the extended dataset objects. The most common error (4.8%) occurred due to faulty identification of lateral grasp for small objects.

Artificial perception grasp classification algorithm results are presented in Figure 6. From 42 images presenting different occurrences of lateral grasp in the testing dataset, 36 were accurately classified. For one object with a handle, palmar grasp was selected due to an error in object handle identification and for 4 objects pinch grasp was selected due to the strict threshold for small object classification, which was optimized based on a different object dataset (training). In one image of an object that should be grasped with lateral grasp color segmentation algorithm detected no objects. Except for the errors on the images of objects that should be grasped with lateral grasp, only one image representing palmar grasp was wrongly classified as lateral grasp and for one image representing pinch grasp no object was detected. Total error of the grasp classification was less than 10%, therefore making the proposed classifier comparable with the grasp type detection algorithms proposed in previous computer vision systems for automatic selection of grasp [25, 26]. The advantage of the Kinect-based approach is additional information about the object size, position and orientation, and the corresponding real world coordinates which can be used to fine tune the stimulation parameters.

**Figure 7:**
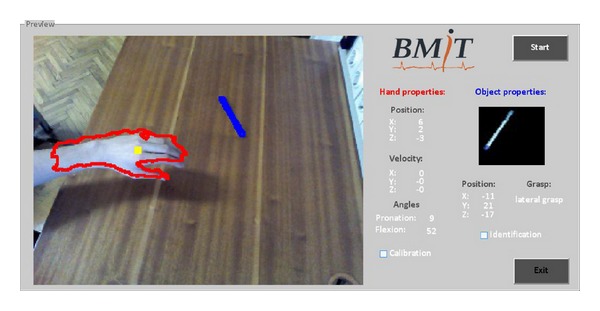
GUI of the MATLAB artificial perception software that presents feedback from the real-time hand tracking with orientation estimation and electrical stimulation control results. The hand position and orientation are tracked continuously in real time (denoted red), while the object model (blue) is determined only once for each object. In this example, the user reaches for the pen placed horizontally on the table surface. The system recognized the object, determined the grasp parameters, and automatically updated the stimulation parameters, and this resulted in a small lateral grasp and adequate hand pronation.

During the real-time evaluation test, artificial perception system outputs (i.e., hand position and orientation estimation, object identification, position estimation, and grasp type selection) that are used to update the stimulation parameters were visually inspected with the custom graphical interface developed in the MATLAB software (Figure 7). Average execution speed of the presented image processing methods for hand tracking and orientation estimation was around 100 ms (Intel Core i5 3337U @ 1.8 GHz, 4 GB of RAM, NVidia GeForce 740 M). These time requirements are sufficient for the timely update of the stimulation parameters even if we account for the delay of Bluetooth communication with the stimulator. The time needed for object identification and grasp classification is longer (<5 s) as it includes computationally expensive operations. However, this does not affect the overall performance since it is done only once for each object that appears in the field of view of the camera. In other words, since only nonmoving objects are used in the therapy assisted by FES, this algorithm step is not required to run in real time during exercise, that is, during the reaching phase of grasping, and can be called only when the object choice has been changed.

## 4. Discussion

In this paper, we have demonstrated the applicability of the artificial perception paradigm for the generation of signals for automatic selection of grasp modality needed for control of FES assisted reaching and grasping. Microsoft Kinect can be used for tracking of hand position and orientation in real time. The presented computer vision algorithm identifies objects and classifies the grasp type with accuracy above 90%. Due to the distinctively predefined system setup we suggest that it can be easily transferred to the clinical environment and tested with patients, specifically as there is no risk for the patients even if the system fails to select the appropriate grasping strategy. The results indicate that this could speed up the accomplishment of tasks during the therapy session and improve overall performance of functional electrical therapy. This hypothesis, however, needs to be verified in future clinical studies.

Our results imply that the presented artificial perception system is not limited to the predefined object set thanks to the generalization feature of the grasp classification algorithm. This feature enables the patient to choose which objects he/she would like to use in the exercise and consequently could increase the training motivation.

This object generalization property of the proposed algorithm also implies that the presented artificial perception system has a very good potential for home use. Thanks to relatively low cost of Kinect camera and simple system setup requirements, that is, a table with a few objects, the demands would mostly come down to the stimulation system. This system should allow easy electrode positioning and selective activation of desired functions. The INTFES system (Tecnalia Research and Innovation, San Sebastian, Spain) with custom designed multipad electrodes and automatic calibration algorithms is one system that already meets most of these requirements [9, 10]. However, the transfer of this technology from a clinical environment to home requires clinical trials to demonstrate the overall benefit of automatic selection of FES assisted grasping protocol compared with conventional hand triggering. Results presented here demonstrate the prospects of using a calibrated RGB-D camera pair to automatically control the FES system during the reach and grasp exercises.

The applicability of the presented computer vision algorithm is not limited to automatic control during the prehension phase and can also be adapted to work in the closed loop control mode. It is important to note that in this paper we have only addressed the prospect of computer vision in automatic control of FES system in the first two stages of the natural hand movements, that is, prehension and grasping. Object manipulation and releasing have been addressed via preprogrammed stimulation patterns. Control of these functional task phases could be further refined by employing a hand tracking algorithm in order to classify and recognize each movement phase and thus automatically select the appropriate stimulation pattern by implementing temporal and spatial synergies.

Presented methodology can also find use in rehabilitation of upper extremities in the form of augmented reality system [44] and automated assessment of the rehabilitation process [45], as part of an intelligent environment that can support and guide older adults with dementia during completion of activities of daily living [46], or for automatic control of a prosthetic hand [21–23]. Future advancements of technology, especially in manufacturing electronic components including cameras, GPUs, and CPUs, will provide more accurate data, enable faster processing, and solve the problem of system portability, thus expanding the possibilities of artificial perception systems in neurorehabilitation [7].

## Figures and Tables

**Table 1:** Correct grasp type for the objects from the image dataset used for setting the classification parameters.

Object	Bottle	Mug	Coffee cup	Pencil	Spoon	Cell phone	CD case	Tennis ball
Grasp type	Palmar	Palmar/lateral	Palmar/lateral	Lateral	Lateral	Pinch	Pinch	Spherical
